# Effects of Color Modifier on Degree of Monomer Conversion, Biaxial Flexural Strength, Surface Microhardness, and Water Sorption/Solubility of Resin Composites

**DOI:** 10.3390/polym13223902

**Published:** 2021-11-11

**Authors:** Pipop Saikaew, Patchara Phimolthares, Pheeratas Phitakthanaakul, Panthira Sirikul, Suwannee Mekrakseree, Piyaphong Panpisut

**Affiliations:** 1 Department of Operative Dentistry and Endodontics, Faculty of Dentistry, Mahidol University, Bangkok 10400, Thailand; pipop.sai@mahidol.ac.th; 2 Faculty of Dentistry, Mahidol University, Bangkok 10400, Thailand; patchara.phimolthares@gmail.com (P.P.); aomsubbpheeratas@gmail.com (P.P.); pd.panthira@gmail.com (P.S.); suwannee.mekrak@gmail.com (S.M.); 3 Division of Restorative Dentistry, Faculty of Dentistry, Thammasat University, Pathum Thani 12120, Thailand; 4 Thammasat University Research Unit in Dental and Bone Substitute Biomaterials, Thammasat University, Pathum Thani 12120, Thailand

**Keywords:** resin composite, color modifier, degree of monomer conversion, biaxial flexural strength, surface microhardness, water sorption, water solubility, tetracycline-induced discoloration

## Abstract

Color modifiers can be mixed with resin composites to mimic the shade of severely discolored tooth. The aim of this study was to assess the effects of a color modifier on the physical and mechanical properties of a resin composite. The composite was mixed with a color modifier at 0 wt% (group 1), 1 wt% (group 2), 2.5 wt% (group 3), or 5 wt% (group 4). The degree of monomer conversion (DC) was examined after light curing for 20 or 40 s. Biaxial flexural strength (BFS)/modulus (BFM), surface microhardness (SH), and water sorption (W_sp_)/solubility (W_sl_) were also tested. The DC of group 1 was significantly higher than that of groups 3 and 4. The increase in curing time from 20 to 40 s increased the DC by ~10%. The BFS, BFM, W_sp_, and W_sl_ of all the groups were comparable. A negative correlation was detected between the concentration of color modifier and the BFS and DC, while a positive correlation was observed with W_sp_. In conclusion, the color modifier reduced the DC of composites, but the conversion was improved by extending the curing time. The increase in color modifier concentration also correlated with a reduction in strength and the increase in the water sorption of the composites.

## 1. Introduction

The systemic administration of tetracycline during skeletal and tooth development leads to the deposition of the drug into the tissues, causing irreversible intrinsic discoloration [1]. The severity of tetracycline-induced tooth discoloration varies from yellow to dark brown, which is a major challenge in restorative dentistry. A common method for managing the lesions or masking the discolored teeth is the use of indirect veneers [2]. The technique generally requires the removal of the tooth surface, followed by the placement of a desirable shade of ceramic to mask the underlying discoloration. The placement of ceramic veneers provides excellent esthetic outcomes [3], but the technique is invasive and requires great experience from the operator [4].

**Figure 1:**
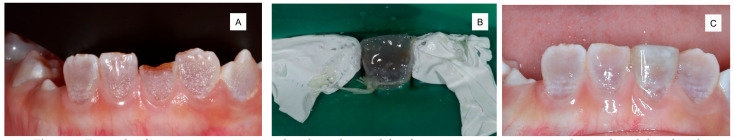
Example of using a composite mixed with a color modifier for restoring a severe tetracycline-induced discolored tooth. (**A**) A patient willing to restore the fracture of the lower left central incisor using direct resin composite; (**B**) resin composite mixed with the color modifier (grey shade), which was used to mimic the discolored dentin; (**C**) final outcome of the composite restoration that exhibits the natural appearance of the discolored tooth.

A minimally invasive approach to restoring tetracycline-induced tooth discoloration is the use of direct resin composites [5]. Additionally, the use of direct composites to manage poor esthetics in anterior teeth was facilitated by the substantial improvement in the physical and mechanical properties of resin composites. However, the shade of most commercial composites is unable to mimic the discolored tooth. The application of light-cured characterizing materials or color modifiers under or between the incremental layers of composite may help to mask the discoloration and produce a natural appearance or desirable restoration shade [6]. Color modifiers consist of light-curable, low-viscosity methacrylate monomers, colorants, and pigments that are available in various colors, such as brown, black, red, or white. The materials contain a low filler content to aid the flowability and adaptation to the surface. The purpose of using a color modifier is to mimic the shade, natural appearance, or characteristics of the tooth [7,8]. However, a study showed that the placement of color modifiers between the composite layers reduced the cohesive strength of the composite, which may affect the longevity of the restoration [7]. Another minimally invasive and simplified method to restore a tetracycline-induced discolored tooth is the use of a composite-mixed color modifier to mimic the shade of the discolored tooth (Figure 1).

The incorporation of a color modifier into composites may reduce the physical and mechanical properties of the materials. The dark pigments from the color modifier may reduce the light transmission, which could decrease the degree of monomer conversion in the materials [9]. The low conversion may reduce the polymer cross-linking and rigidity of the polymer network. This may subsequently promote water sorption/solubility and the release of monomers from the material [10,11]. Furthermore, the identified unreacted monomers of composites have been shown to induce cytotoxic, genotoxic, mutagenic, carcinogenic, and allergenic effects in the in vitro studies [12]. The darker composites reached the highest polymerization after light curing, slower than the composites with lighter shades, which led to a low degree of monomer conversion [13,14]. Additionally, the darker-shade composites tend to absorb more light, and require a longer exposure time compared with lighter-shade composites [9]. Furthermore, the incorporation of low-molecular-weight monomers from the color modifier may decrease the mechanical properties of the composites [15,16], which could potentially reduce the longevity of the restoration.

At present, the evidence explaining the effect of the incorporation of a color modifier on the physical and mechanical properties of resin composites is limited. The aim of the current study was, therefore, to assess the effect of the incorporation of a color modifier on the degree of monomer conversion (after light-curing for 20 or 40 s), surface microhardness, biaxial flexural strength/modulus, and the water sorption/solubility of the composite material. The null hypothesis was that the addition of a color modifier at different concentrations would have no significant effect on the physical/mechanical properties of the material.

## 2. Materials and Methods

### 2.1. Materials Preparation

**Figure 2:**
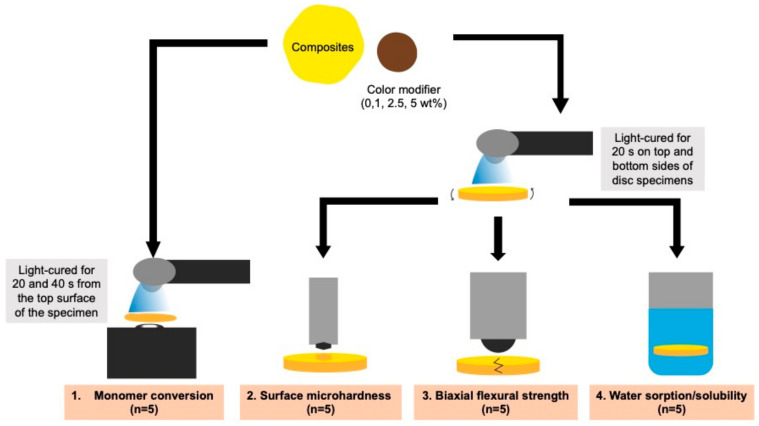
Schematic illustrating the methods used in the current study.

A commercial resin composite (Harmonize shade A3, Kerr Corporation, Orange, CA, USA) was mixed with a color modifier (Kolor Plus, Kerr Corporation, Orange, CA, USA) at 0 wt% (group 1 or control), 1 wt% (group 2), 2.5 wt% (group 3), and 5 wt% (group 4). The materials were weighed using a four-figure balance and hand-mixed within 20 s in a dark box. The compositions of the commercial materials are presented in Table 1. A schematic explaining the protocol used in the current study is presented in Figure 2.

### 2.2. Degree of Monomer Conversion (DC)

The DC was measured using an attenuated, total reflection Fourier-transform infrared spectrometer (ATR-FTIR, Nicolet i5, Thermo Fisher Scientific, Waltham, MA, USA) (*n* = 5) [17]. The composite and color modifier were weighed and hand-mixed within 20 s. The mixed paste was placed in the metal ring (1-mm thickness) on the ATR diamond. The paste was covered and pressed with an acetate sheet so that the thickness of the composites was fixed at 1 mm. They were light-cured using an LED light-curing unit (irradiance of 1200 mW/cm^2^, SmartLite Focus Pen Style, DENTSPLY Sirona, York, PA, USA) from the top surface for 20 and 40 s (Figure 1). The curing time of 20 or 40 s is clinically relevant and commonly used in curing protocols for resin composites [18]. FTIR spectra were obtained in the region of 700–1800 cm^–1^ at the bottom of the specimen before and after curing. The test was conducted at room temperature (25 ± 1 °C). The DC (%) of the specimen was then calculated, using the following equation:

(1) $$\mathrm{DC}=\frac{100\left(\Delta A_{0}-\Delta A_{t}\right)}{\Delta A_{0}}$$

where $\Delta A_{0}$ and $\Delta A_{t}\;$ represent the absorbance of the C-O peak (1320 cm^–1^) above the background level at 1335 cm^–1^ before and after curing at time *t*, respectively. The peak at 1320 cm^–1^ [ν (C-O)] of the methacrylate group was used to calculate the DC due to the lower variation in the result compared to that obtained from the peak at 1636 cm^–1^ [ν (C=C)] [19].

### 2.3. Surface Microhardness (SH)

Disc specimens (*n* = 5) were prepared according to the previous section. They were immersed in 10 mL of deionized water at 37 °C for 24 h before the test. The Vickers surface microhardness of the specimens was tested using a microhardness tester (FM-800, Future-Tech Corp, Kanagawa, Japan) at room temperature (25 ± 1 °C), with an indenter load of 50 g for an indentation time of 15 s [20,21]. The results were recorded as Vickers hardness number (VHN). The obtained hardness value of each specimen was the average of values measured from four areas on the surface.

### 2.4. Biaxial Flexural Strength (BFS) and Modulus (BFM)

The composites and color modifier were weighed and mixed within 20 s. The mixed pastes were loaded into a metal circlip (10 mm in diameter and 1 mm in thickness, Springmasters, Redditch, UK). The specimens were covered with an acetate sheet and glass slaps on the top and bottom surfaces. They were light-cured using the LED light-curing unit for 20 s on the top and bottom sides to produce disc specimens (Figure 1). The specimens were left at room temperature for 24 h to allow the process to complete. Then, the specimens were removed from the circlip and any excess was trimmed. They were placed in tubes containing 5 mL of deionized water. The tubes were incubated at 37 °C for 24 h before the test.

The biaxial flexural strength (BFS) test was conducted at room temperature (25 ± 1 °C). The disc specimen was placed on a ball-on-ring testing jig under a mechanical testing frame (AGSX, Shimadzu, Kyoto, Japan). The load cell (500 N) was applied on the jig at a crosshead speed of 1 mm/min until the specimen was fractured. The load at failure was then recorded. The BFS (Pa) was then calculated according to the following equation [22]:

(2) $$\mathrm{BFS}=\frac{F}{d^{2}}\left\{\left(1+v\right)\left[0.485\ln\left(\frac{r}{d}\right)+0.52\right]+0.48\right\}$$

where F is the load at failure (N), d is the specimen’s thickness (m), r is the radius of circular support (mm), and v is Poisson’s ratio (0.3). Then, the biaxial flexural modulus (BFM, Pa) was obtained using the following equation [23]:

(3) $$\mathrm{BFM}=\left(\frac{\Delta H}{{\Delta W}_{c}}\right)\times \left(\frac{\beta_{c}d^{2}}{q^{3}}\right)$$

where $\frac{\Delta H}{{\Delta W}_{c}}$ is the rate of change of the load with regards to the central deflection versus the gradient of the force–displacement curve (N/m), $\beta_{c}$ is the center deflection junction (0.5024), and q is the ratio of the support radius to the radius of the disc.

### 2.5. Water Sorption (W_sp_) and Water Solubility (W_sl_)

Disc specimens were prepared (*n* = 5). They were placed in the first desiccator with a controlled temperature of 37 ± 1 °C for 22 h. Then, the specimens were moved to the second desiccator with a controlled temperature of 25 ± 1 °C for 2 h. The mass of the specimens was then measured using a four-figure balance. These procedures were repeated until a constant mass (conditioned mass, m_1_) was obtained.

The specimens were then placed in a tube containing 10 mL of deionized water. They were placed in an incubator with a controlled temperature of 37 ± 1 °C for 7 days. Then, the specimens were removed and blotted dry. The mass of the specimens was recorded after 7 days (m_2_).

The specimens were then reconditioned following the procedure described above for m_1_. The reconditioning was repeated until a constant mass was obtained (m_3_). The water sorption (W_sp_, g/m^3^) and water solubility (W_sl_, g/m^3^) of the materials were calculated using the following equations [24]:

(4) $$W_{\mathrm{sp}}=\frac{m_{2}-m_{3}}{v}$$

(5) $$W_{\mathrm{sl}}=\frac{m_{1}-m_{3}}{v}$$

where m_1_ is the conditioned mass of the specimen (g), m_2_ is the mass of the specimen after immersion in water for 7 days (g), m_3_ is the reconditioned mass of the specimen after immersion in water (g), and v is the volume of the specimen (m^3^).

### 2.6. Statistical Analysis

The numerical data presented in the current study are means ± SD. The data were analyzed using Prism 9.2 (GraphPad Software LLC., San Diego, CA, USA). The normality of the data was assessed using the Shapiro–Wilk test. Then, data were analyzed using a one-way ANOVA, followed by Tukey’s multiple comparisons. Additionally, the difference in DC upon curing for 20 or 40 s was examined using a repeated-measures ANOVA and Tukey’s post hoc multiple comparisons test. Pearson’s correlation analysis was additionally performed to examine the correlation between the concentration of color modifier and the DC, SH, BFS/BFM, W_sp_, and W_sl_ of composites. All *p*-values lower than 0.05 were considered statistically significant. Power analysis was performed using G^*^Power 3.1 (University of Dusseldorf, Germany) [25] based on the results from previously published studies [20,21,22]. The results from G^*^Power suggested that five samples per group were required to obtain a power greater than 0.95 in a one-way ANOVA (α = 0.05).

## 3. Results

### 3.1. Degree of Monomer Conversion (DC)

**Figure 3:**
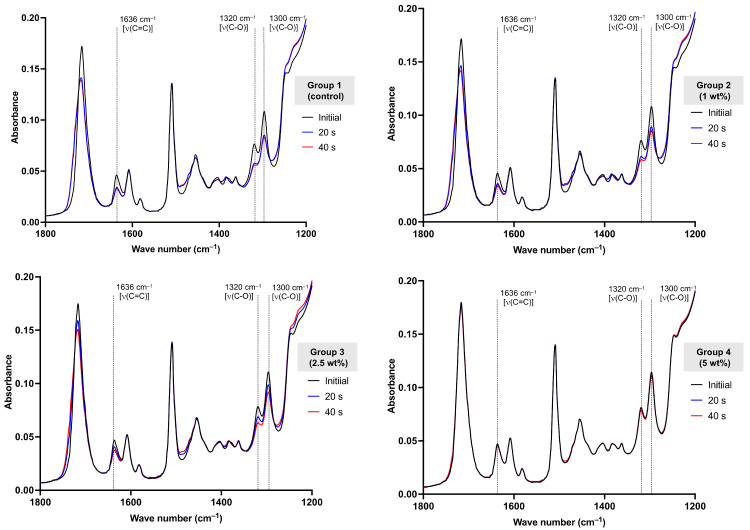
Representative FTIR spectra of composites before and after curing for 20 and 40 s from each group.

A reduction in the peak at 1320 cm^–1^ was observed after light-curing (Figure 3). The reduction was increased upon extension of the light-curing time (20 to 40 s). However, the reduction in the peak was less evident in groups 3 and 4. Group 1 exhibited the highest DC after curing for 20 (42.8 ± 1.6%) and 40 s (49.1 ± 1.0%) compared with the other groups (Table 2). Group 4 showed the lowest degree of monomer conversion at 20 (3.3 ± 3.7%) and 40 s (7.9 ± 7.6%).

**Figure 4:**
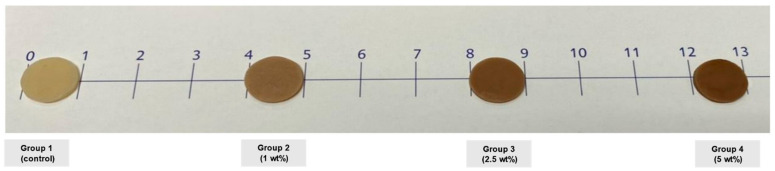
Appearance of specimens after mixing with color modifier.

**Figure 5:**
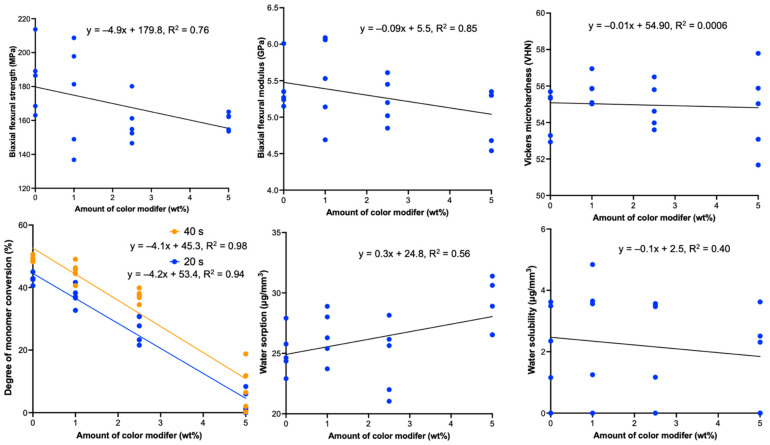
The linear regression of each property versus the amount of the color modifier.

The appearance of the specimens in each group after light-curing is presented in Figure 4. Higher concentration of the color modifier led to a darker shade of the specimens. The conversion in group 1 at 20 and 40 s was not significantly different from that of group 2 (20 s, 37.3 ± 3.2%; 40 s, 45.2 ± 3.1%) (*p >* 0.05). The conversion in all groups after being light-cured for 40 s was significantly higher than that at 20 s (*p* < 0.05). Additionally, a negative correlation was detected between the concentration of color modifier and the degree of monomer conversion at 20 and 40 s (*p* < 0.01) (Figure 5).

### 3.2. Surface Microhardness

The surface microhardness values obtained from group 1 (54.5 ± 1.3 VHN), group 2 (55.8 ± 0.8 VHN), group 3 (54.9 ± 1.2 VHN), and group 4 (54.7 ± 2.4 VHN) were comparable (*p* > 0.05) (Table 2). Additionally, no correlation was detected between the concentration of color modifier and surface microhardness (*p* = 0.7686) (Figure 5).

### 3.3. Biaxial Flexural Strength (BFS) and Modulus (BFM)

**Figure 6:**
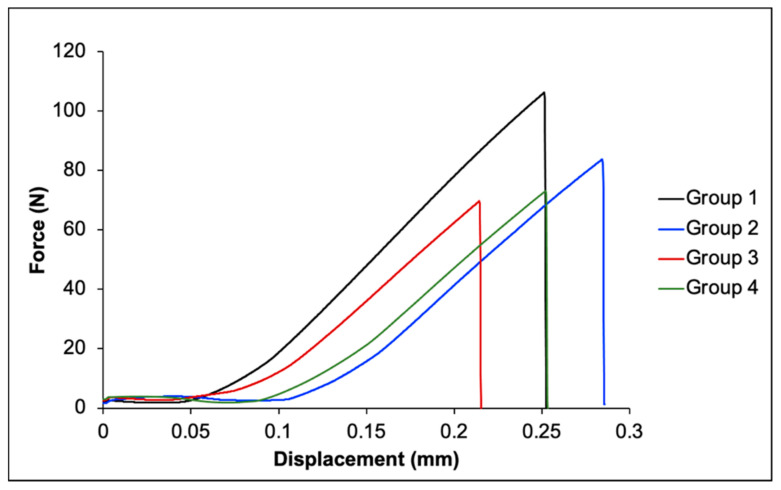
Representative force–displacement diagram from BFS testing.

An increase in force was observed in all groups following the increase in displacement (Figure 6). The highest and lowest BFS values were obtained from group 1 (184.2 ± 20.0 MPa) and group 3 (159.1 ± 12.9 MPa), respectively (Table 2). No significant differences were detected among the BFS values obtained from group 1, group 2 (174.7 ± 31.0 MPa), group 3, and group 4 (159.6 ± 5.1 MPa) (*p* > 0.05). For BFM, the highest mean value was observed in group 2 (5.5 ± 0.6 GPa), followed by group 1 (5.4 ± 0.4 GPa), group 3 (5.2 ± 0.4 GPa), and group 4 (5.0 ± 0.4 GPa). However, the results were not significantly different (*p* > 0.05). Additionally, a negative correlation was detected between the concentration of color modifier and BFS (*p =* 0.048). However, no correlation between BFM and the level of color modifier (*p =* 0.110) (Figure 5).

### 3.4. Water Sorption (W_sp_) and Water Solubility (W_sl_)

The highest and lowest mean W_sp_ were observed in group 4 (28.8 ± 2.3 μg/mm^3^) and group 3 (24.6 ± 3.0 μg/mm^3^), respectively (Table 2). Additionally, the highest and lowest mean W_sl_ were observed in group 2 (2.7 ± 1.7 μg/mm^3^) and group 4 (1.7 ± 1.4 μg/mm^3^), respectively. No significant differences were detected in W_sp_ and W_sl_ among (*p* > 0.05). Furthermore, no correlation was observed between the concentration of color modifier and W_sl_ (*p =* 0.5275). However, a positive correlation was detected between the concentration of color modifier and W_sp_ (*p =* 0.0487).

## 4. Discussion

The aim of the current study was to assess the effect of using different concentrations of color modifier on the physical and mechanical properties of the composites. The use of the color modifier significantly reduced the degree of monomer conversion of the composites. Hence, the null hypothesis was partially rejected. It should be mentioned that the current study is an in vitro study. Hence, the related clinical significance should be carefully interpreted.

The degree of monomer conversion is primarily governed by the chemical structures of monomers [26], the concentration and type of photoinitiators [27], translucency and shade of materials [28], and the irradiance of light-curing units [29]. A high degree of monomer conversion after light curing may generally help ensure good physical and mechanical properties in the restored composites [30]. This may additionally decrease the risk of releasing toxic, unreacted monomers [31,32]. In general, the DC, after a sufficient light-curing time of conventional composites, ranged from 50 to 70% [18,30].

**Figure 7:**
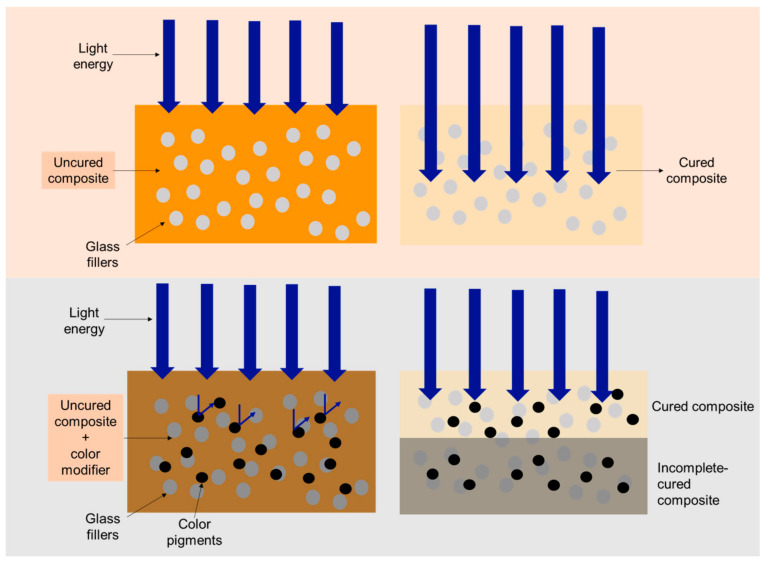
The addition of color modifiers reduced the degree of monomer conversion of resin composites.

The increase in color modifier concentration significantly reduced the DC of the composites on the inner surface. The current study showed that composites with an added color modifier of greater than 1 wt% exhibited DC values lower than 40%, even after being light-cured for 40 s. It is known that the maximum curing depth in light-activated free-radical polymerization is limited by the attenuation of curing light. This could be explained using Beer–Lambert’s law [33,34] (Equation (6))

(6) $$I={\;I}_{0}e^{-\gamma d}$$

where I and $I_{0}$ are the light intensity at depth d and light intensity entering the specimen surface, respectively. γ is the Naperial absorption coefficient of the medium. The reduction in light intensity in the composites upon the addition of a color modifier may be due to the light absorption and the increase in light scattering caused by fillers and other additives [34]. This could subsequently lead to a limited curing depth and the production of free radicals in the materials. Additionally, the transmission of light energy into the composites may be diminished by the increase in darkness of the composite shade [35] (Figure 7). Furthermore, dark pigments of a color modifier may block the light penetration or increase the light scattering due to the increase in refractive index mismatch in the composites [9,36,37]. It was reported that the color pigments may act as the light-scattering centers, which could reduce light penetration into the composites in a dose-dependent manner [38].

The reduction in DC may lead to the release of unreacted monomers from the composites. Future work should, therefore, investigate the monomer elution using HPLC. The results of the current study also suggest that the DC of composites mixed with the color modifier was increased by ~10% after extending the light-curing time from 20 to 40 s. This could be due to the increase in the radiant exposure, which could promote the production of free radicals [39] to enhance the DC of the materials [40,41]. Another method to enhance the polymerization could be the use of a high-irradiance light-curing unit [42].

Negative correlations were detected in the concentration of color modifier versus water sorption and biaxial flexural strength. It is known that water sorption is generally associated with the DC, the hydrophilicity/hydrophobicity of the polymers, and the structure of the polymer network [43]. The reduction in DC due to the addition of a color modifier may reduce the polymer cross-link of the composites. This may subsequently decrease the rigidity of the polymer network and increase water sorption into the materials. Additionally, the primary methacrylate monomer of the color modifier is triethylene glycol dimethacrylate (TEGDMA). It was demonstrated that poly-TEGDMA absorbed more water than other dimethacrylate polymers [44]. This could be due to the heterogenicity of poly-TEGDMA, which contains microporosities or clusters inside the polymer network. The space created between the clusters may accommodate a large quantity of water. Additionally, the high flexibility of poly-TEGDMA, due to its low molecular weight (TEGDMA monomer = 286.3 g/mol), may allow for swelling of the polymer chain due to water. The adsorbed water can act as a plasticizer that increases polymer plasticization, thus reducing the strength of the composites [45,46,47].

It should be mentioned that no significant differences were detected in the strength, surface microhardness, and water sorption/solubility of the composites in each group. This could be due to the fact that the composite specimens were light-cured on both sides following the protocol used in the BS ISO 4049 (Dentistry—polymer-based restorative materials) [24]. This may enhance the physical and mechanical strength of the specimens. Therefore, the main limitation of the current study was that the specimen preparation did not represent the actual clinical situation, where the composites can only be light-cured on the outer surface. Therefore, future work may need to prepare for specimens to be light-cured from only one side to mimic the clinical reality.

## 5. Conclusions

Within the limits of the current in vitro study, it is possible to draw the following conclusions:

− - The composites containing different concentrations of color modifier (1, 2.5, or 5 wt%) exhibited no significant differences in biaxial flexural strength/modulus, surface microhardness, water sorption, and water solubility;
− - The increase in color modifier concentration was correlated with a reduction in the degree of monomer conversion and the biaxial flexural strength of the composites. Additionally, the increase in color modifier concentration was correlated with an increase in the water sorption of the materials;
− - The increase in light-curing time from 20 to 40 s significantly enhanced the degree of monomer conversion of the composites that were mixed with the color modifier.

## Figures and Tables

**Table 1:** The composition of the commercial materials used in the current study. The exact amount is not provided by the manufacturer.

Materials	Composition	Suppliers
Harmonize (Shade A3)	- Poly(oxy-1,2-ethanediyl) and α,α’-[(1- methylethylidene)di-4,1-phenylene]bis[ω-[(2- methyl-1-oxo-2-propen-1-yl)oxy] (25–50 wt%) - 3-trimethoxysilylpropyl methacrylate (<5 wt%) - 2,2’-ethylenedioxydiethyl dimethacrylate (<3 wt%) - Barium glass	Kerr Corporation, Orange, CA, USA
Kolor Plus (Shade brown)	- Silanated barium borosilicate glass (30–60 wt%), - Triethylene glycol dimethacrylate (< 10 wt%) - Fume silica treated (1–5 wt%) - Fumed silica (1–5 wt%) - Titanium dioxide (1–5 wt%) - 2-ethylhexyl-4-(dimethylamino) benzoate (<1 wt%) - 2-hydroxy-4-methoxybenzophenone (<1 wt%) - 2,6-di-(tert-butyl)-4-methylphenol (<1 wt%)	Kerr Corporation, Orange, CA, USA

**Table 2:** The results (mean and SD) from each group. The same lowercase letters indicate significant differences (*p* < 0.05) between groups in the same column. The same uppercase letters indicate significant differences (*p* < 0.05) in DC in the same group after curing for 20 or 40 s.

Materials/Properties	1. DC 20 s (%)	2. DC 40 s (%)	3. BFS (MPa)	4. BFM (GPa)	5. SH (VHN)	6. W_sp_ (μg/mm^3^)	7. W_sl_ (μg/mm^3^)
Group 1 (Control group)	42.8 (1.6) a,b,A	49.1 (1.0) a,b,A	184.2 (20.0)	5.4 (0.3)	54.2 (1.7)	25.1 (1.9)	2.1 (1.5)
Group 2 (1 wt%)	37.3 (3.2) c,d,B	45.2 (3.1) c,d,B	174.7 (31.0)	5.5 (0.6)	55.8 (0.8)	26.5 (2.1)	2.7 (1.7)
Group 3 (2.5 wt%)	26.8 (4.2) a,c,e,C	37.3 (2.0) a,c,e,C	159.1 (12.9)	5.2 (0.3)	54.9 (1.2)	24.6 (3.0)	2.3 (1.5)
Group 4 (5 wt%)	3.3 (3.7) b,d,e,D	7.9 (7.6) a,c,e,D	159.6 (5.1)	5.0 (0.4)	54.7 (2.4)	28.8 (2.3)	1.7 (1.4)
*p-*value from correlation analysis	<0.0001	<0.0001	0.0476	0.0887	0.7686	0.0487	0.5275

## Data Availability

The datasets generated and/or analyzed during the current study are available from the corresponding author on reasonable request.
